# Differences and Commonalities of Electrical Stimulation Paradigms After Central Paralysis and Amputation

**DOI:** 10.1111/aor.15017

**Published:** 2025-05-02

**Authors:** Thomas Stieglitz, Ines Bersch, Natalie Mrachacz‐Kersting, Cristian Pasluosta

**Affiliations:** ^1^ Laboratory for Biomedical Microtechnology, Department of Microsystems Engineering‐IMTEK University of Freiburg Freiburg Germany; ^2^ BrainLinks‐BrainTools//IMBIT University of Freiburg Freiburg Germany; ^3^ Bernstein Center Freiburg University of Freiburg Freiburg Germany; ^4^ International FES Centre Swiss Paraplegic Center Nottwil Switzerland; ^5^ Department of Sports and Sport Sciences University of Freiburg Freiburg Germany

**Keywords:** BCI, control, FES, LTP, prostheses, sensorimotor assessment, sensory feedback, spinal cord injury

## Abstract

**Background:**

Patients with spinal cord injury (SCI) or with severe brain stroke suffer from life‐lasting functional and sensory impairments. Other traumatic injuries such as limb loss after an accident or disease also affect motor function and sensory feedback and impair quality of life in those individuals. Invasive and non‐invasive functional electrical stimulation (FES) is a well‐established method to partially restore function and sensory feedback of paralyzed and phantom limbs. It is also a supporting technology for the rehabilitation of the neuromuscular system and for complementing assistive devices.

**Methods:**

This work reviews the current state‐of‐the‐art of FES as a technology for restoring function and supporting rehabilitation therapy and assistive devices.

**Results:**

Electrodes, electrical stimulation, use of brain signals for rehabilitation and control, and sensory feedback are covered as parts of the whole. A perspective is given on how clinical and research protocols developed for patients with SCI and brain injuries can be translated to the treatment of patients with an amputation and vice versa. We further elaborate on how motor learning strategies with quantitative electrophysiological and kinematic measurements may help caregivers in the rehabilitation process. Insights from practitioners (collected during a workshop of the IFESS 2025) have been integrated to summarize common needs, open questions, and challenges.

**Conclusions:**

The information from the literature and from practitioners was integrated to propose the next steps towards establishing common guidelines and measures of FES in clinical practice towards evidence‐driven treatment and objective assessments.

## Graphical abstract

Invasive and non‐invasive functional electrical stimulation (FES) is a well‐established method to partially restore function after paralysis and deliver sensory feedback after amputation. This work summarizes the current state‐of‐the‐art of FES for restoring function, supporting rehabilitation therapy, and assistive devices. We suggest future directions to strengthen this methodology in practice.

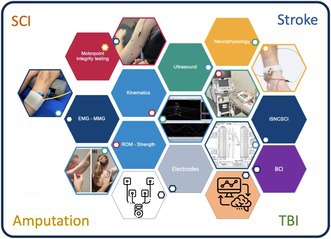

## 1 Introduction

**FIGURE 1:**
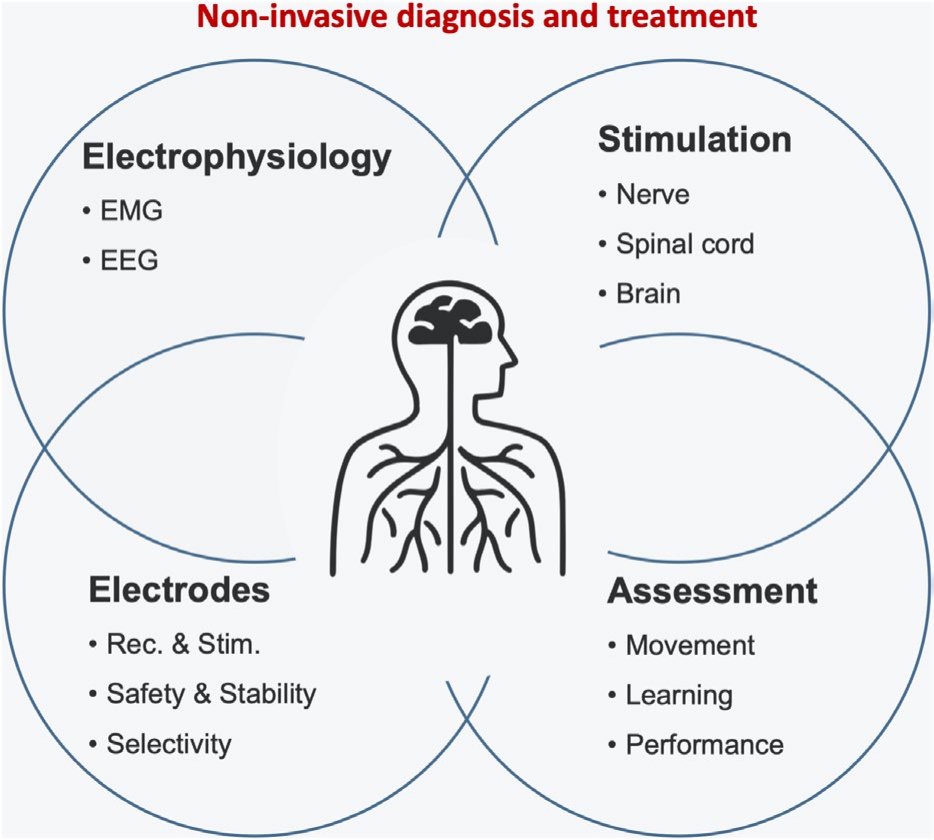
Aspects of comprehensive and objective assessment in sensory and motor rehabilitation. [Color figure can be viewed at wileyonlinelibrary.com]

Injuries at the spinal cord or the brain pose life lasting functional and sensory impairments. Limb loss after trauma or disease also affects sensorimotor function and disrupts quality of life in those individuals. Functional electrical stimulation (FES) utilizing invasive [1, 2, 3, 4] and non‐invasive technology [5] is an area of research aimed to partially restore function and sensory feedback in these populations. A variety of approaches has been proposed [6] up to first‐in‐human clinical trials to partially restore sensorimotor functions, allowing the control of paralyzed limbs or assistive devices using signals from the central [7] and peripheral [8] nervous system. However, on the long road to clinical practice, promising technological and medical approaches fail to become established business concepts and standardized clinical treatments due to low patient numbers in specific disease conditions, high variability in disease characteristics, long setup times that might extend the “window of opportunity” for optimal treatment and high device and treatment costs. In this review article, we focus mainly on non‐invasive applications which can be applied “almost immediately” to patients and be transferred into clinical settings, including neurorehabilitation centers, independent practitioners, and potentially at‐home care (Figure 1). Approaches in which implantable systems are used [9] are even more complex and comprehensive expert knowledge from various disciplines ranging from fundamental neuroscience over engineering to regulatory affairs needs to be combined. Inclusion of these invasive devices in a comprehensive discussion would go far beyond the scope of this review. Here, we provide a perspective on how to integrate non‐invasive approaches into clinical and research protocols to diagnose and treat patients with spinal cord injury (SCI) and brain injury. We further discuss how these protocols can be translated to the treatment of patients after amputation of a limb. We advocate that motor learning strategies with quantitative measurements may provide caregivers with important tools for the caring of these populations.

Electrical measurements from the brain and muscles are often complemented by magnetic and electrical stimulation to better detect and diagnose the pathophysiological status of a person, to assess the influence of treatment on learning (i.e., modulation) of muscle activation of the lower extremity, and to quantize performance and its course over therapeutic sessions and beyond (carry over effect). This review presents methods for a comprehensive and objective assessment of outcomes after electrical stimulation. Examples of how and why different aspects should be considered (and why it matters) when performing electrical stimulation as a method for treatment and rehabilitation will be presented. Further, how “other” methods such as brain‐computer interfaces (BCIs), transcutaneous spinal cord stimulation (tSCS), and transcranial magnetic stimulation (TMS) influence the outcome of the treatment will be discussed later in this review. A subjective perspective on why brain signals should be included in clinical practice, on why the actual terminology of SCI classification needs to be applied, on how and where learning takes place (e.g., pathways beyond the pyramidal tract, intersegmental plasticity, short and long‐term potentiation) has been included as well as thoughts on the importance of the chosen stimulation electrodes. At last, we suggest methods to derive more objective informative features during patient assessment.

We have experienced the challenges of efficiently communicating in a multidisciplinary team and learn the language of the other disciplines. This review is based on a workshop held during the Annual Conference of the International Functional Electrical Stimulation Society in the year 2024. It will provide a bird's eye view on four aspects to picture the use cases of electrical stimulation in rehabilitation, including (1) knowledge on electrodes as the key interface towards function, (2) FES‐driven motor learning, (3) the use of brain signals to improve outcomes of FES (and motor learning) in rehabilitation, (4) assessment of sensorimotor output without and with electrostimulation after limb loss. Although these subjects appear to be unrelated at first glance, the following discussion will outline their intersections and synergies, as well as highlight their distinguishing characteristics. The intention is to share thoughts and experiences that have been enriched by the contributions of 13 participants (see details on the workshop, the methods and the participants in the acknowledgements section) during an interactive session as part of the workshop.

The translation between theory and practice, engineering, medicine, and physiotherapy will be considered in suggestions on the way forward as well as an assessment of challenges and opportunities that scientists and practitioners experience in applying established and new methods.

## 2 Electrodes—The Recording and Stimulation Interface

**FIGURE 2:**
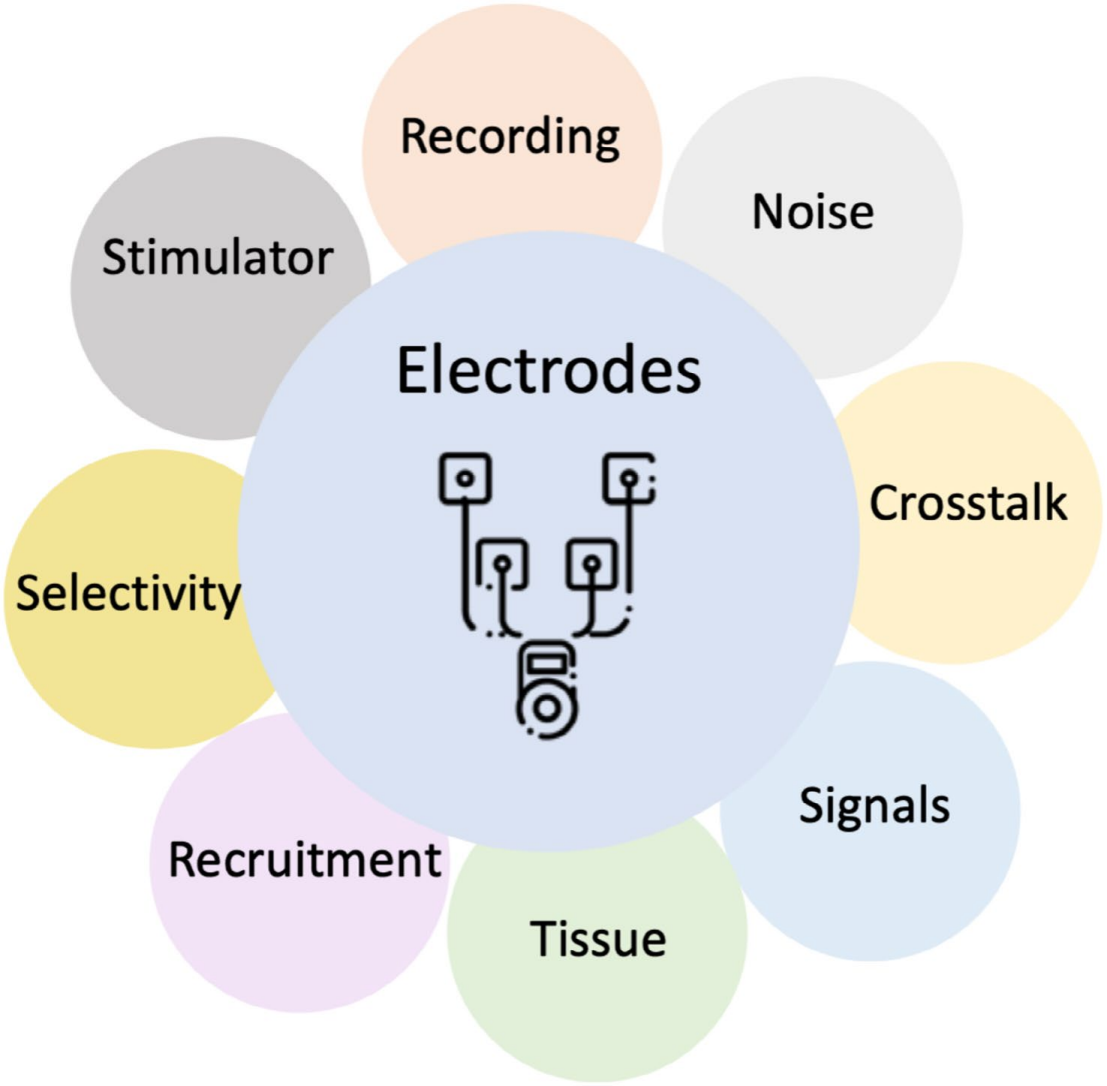
Multiple factors influence safety, performance and longevity of electrodes. [Color figure can be viewed at wileyonlinelibrary.com]

Recording of electrical signals from nerve and muscle cells and electrical stimulation of the nervous system require electrodes as transducers between the target in the body and the electrical and electronic equipment such as cables, connectors, amplifiers, or stimulation circuitry. Electrode development is a vivid topic of research in (neural) engineering and material sciences, which includes both material and device development [10, 11, 12]. Material developments focus on the reduction of noise during the recording of signals and the increase of the amount of charge that can be transferred during electrical stimulation. Device development goes hand in hand with questions from fundamental (preclinical) neuroscience and novel treatment approaches of mostly invasive systems to access the nervous target tissue. The smallest dimensions of the implant and large numbers of contact sites are desirable to access as many independent signal sources or stimulation targets as possible while harming the tissue the least possible. While the generation of knowledge and publications in journals with high impact factors are generally driving factors, these approaches do not always come with the idea of translation into human applications [13]. Sometimes, some devices become standard tools in neuroscience research on rodents' experiments, but only a handful of truly translational research approaches succeed in approved medical devices and become into reimbursement frameworks as priority treatment. The only examples in active implantable medical devices on this level are the cardiac pacemaker and the cochlear implant. All the other approaches are still considered as “last resort” therapies after pharmaceutical medications have failed, e.g., in epilepsy or Parkinson's disease treatment. In most clinical cases using FES, electrodes come as non‐invasive devices to be placed on the skin as close as possible to the electrical source or target. Electrode material, contact to the skin, size, distance between them (e.g., several electrodes on multiple devices or integrated into a single array) and anatomy as well as the recording or stimulation electronics determine their performance. The relation between performance and application must always be considered to guarantee the best possible recording and stimulation (Figure 2).

Engineers describe recording electrode performance [14, 15, 16] by their electrochemical impedance spectrum (EIS). Characterization takes place using sinusoidal electrical signals as input over a broad frequency range. Attenuation of the magnitude (|Z|) and displacement (phase φ) of sinusoidal current through the electrode after the application of a sinusoidal voltage of a small magnitude (from 10 mV to 100 mV) is measured and gives experts insights on which signal frequencies pass and which ones get attenuated at which degree. Multiple factors influence electrode performance [17]. The resistance of the electrode contributes to the (thermal) noise of the electrode overlaying the electrical signals. The size of an electrode and its surface roughness are the first factors determining its impedance. The larger an electrode is, the lower the impedance. The surface acts as a capacitor (Helmholtz capacitance) to the body, with a layer of water with ions (salt and electrolytes) in between. Electrode gels or hydrogels are established materials to mediate this contact to the skin. Silver/silver chloride is used as a material combination to establish, together with the gel, a reliable electrical interface. Sweat glands and hair follicles in the skin let the gel (water with ions) bridge the high resistivity of the epidermis and create a low‐resistance contact to the subcutis. Dry electrodes lack this opportunity and often come with higher noise levels due to a higher contact resistance. Finally, textile electrodes are made of metals, conductive rubbers, and carbon fibers. Hydrogels as well as conjugated polymers (e.g., PEDOT:PSS) [18] surface coatings may also replace the gel. Both (hydro‐)gels and polymer coatings suffer from changing resistances over time since water might evaporate or humidity and sweat might get absorbed, changing the transfer resistance.

Stimulation electrodes [14, 16, 19] need to transfer charge over time (i.e., current) into the tissue without causing electrode corrosion or acidic or thermal burns of the skin. Therefore, the voltage drop over the electrode needs to be as small as possible to limit the generation of Joules heat. The electrochemical reactions at the interface during stimulation need to stay in the reversible range. Material properties and electrochemically active surface area (i.e., roughness) determine how much charge can be transferred. Irreversible processes do not only damage the electrode, but might also lead to pH shifts with succeeding acidic burns. The larger the electrodes are, the more current they can transfer. However, in both recording and stimulation, larger sizes also cover larger areas of the anatomical target and hence record from more sources or stimulate larger populations of nerve and/or muscle fibers. Further, since the body is highly conductive, electrical fields get superimposed, reducing spatial selectivity. This needs to be considered when integrating single electrodes. It can be advantageous with respect to redundancy and tolerance to consider these effects in electrode placement; for example, choose electrode size accordingly. At the same time, it can be disadvantageous if several sources are superimposed and different signal sources are merged in the recorded signal. In the case of stimulation, electrodes can get short‐circuited by sweat or gel if they are too close. In addition, if the area used for stimulation is too small, the excitation threshold may not be overcome, and it will not be possible to achieve maximum motor activation at chemically safe charge injection limits. Careful selection of electrodes is mandatory, and conversation between the product manager of the device company, the clinical engineer, and the health care personnel should not be underestimated as an important asset to achieve robust and reliable results out of diagnosis and treatment.

## 3 Optimized Motor Learning Using FES‐Based Technology

The optimization of motor learning through the utilization of FES‐based technology represents a promising avenue of research. The process of motor learning can be enhanced through the application of assorted FES modalities. These modalities include afferent and efferent stimulation, transcutaneous spinal cord stimulation (tSCS) and paired associative stimulation (PAS). The application of afferent stimulation results in the generation of an action potential at the level of the afferent neuron. In some neurological conditions, it is difficult to determine the sensory threshold due to the lack of sensory feedback. In this case, the motor threshold is determined, and the stimulation intensity is reduced until there is no visible contraction. At higher stimulation intensities, the efferent/motor neuron is depolarized, which is referred to as the motor threshold. tSCS is based on the theory that it activates proprioceptive fibers, which in turn transmit mono‐ and polysynaptic impulses to motoneurons. This form of stimulation has gained attention for its potential to modulate spinal networks and enhance motor function [20, 21]. The antidromic effect is exploited by PAS through the simultaneous stimulation of the motor cortex of the desired region and the associated nerve located in the periphery [22]. All modalities are designed to retrain the nervous system and stimulate a form of learning that restructures the residual parts of the nervous system and promotes new neural connectivity [20]. The aforementioned approaches either influenced the nervous system directly at the neuronal level or indirectly over effects at the muscular level.

The concept of FES can be elucidated through the lens of Hebbian theory. In the post‐injury period, damaged ascending and descending neuropathways continue to deteriorate, which results in the atrophy of paretic and paralyzed muscles. FES can be employed to reinforce the functional connections between the brain and affected muscles by directing adaptive plasticity, activating damaged networks via spared fibers, and inducing neuronal plasticity via cortical neuron‐controlled FES [23]. Furthermore, a demonstrable effect can be observed at the muscular level. The application of FES with the selected parameters can influence and partially reverse neurological muscle alterations [23, 24, 25, 26, 27, 28, 29, 30].

The changes observed in the muscle depending on the type of the underlying injury include disuse and/or denervation atrophy, reduced oxidative and enzymatic activities, a shift in muscle fiber type from type I to type IIX with the emergence of hybrid fibers, changes in muscle morphology such as the transformation into fat and connective tissue, the loss of contractile units, a reduction in muscle strength (low fatigue resistance—negatively correlated with time since injury) and a reduction in vascularization.

**FIGURE 3:**
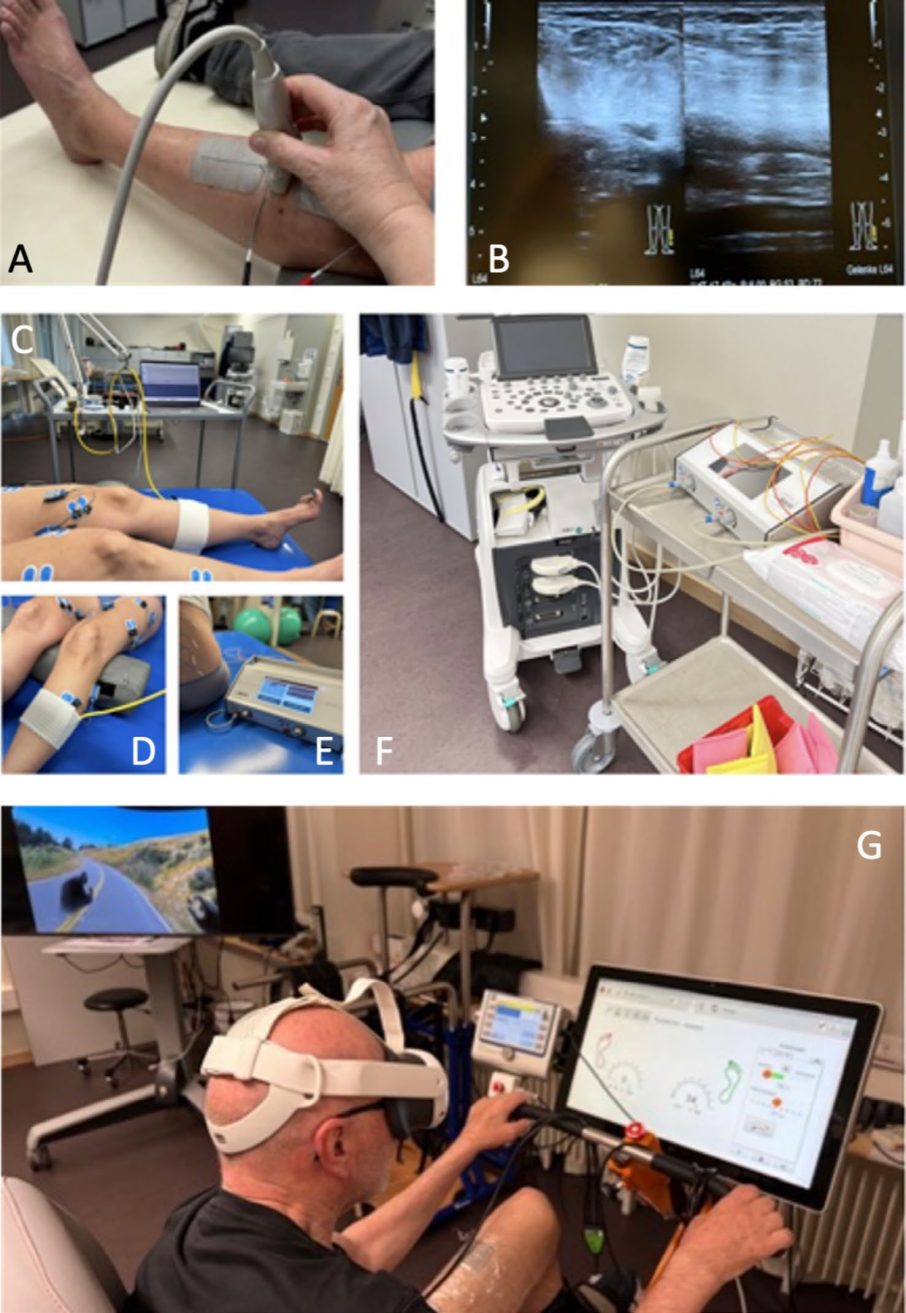
(A) Ultrasound of the tibialis anterior muscle under electrical stimulation with 300 us, 35 Hz and 40 mA. (B) Contracted muscle on the left side, relaxed muscle on the right side. (C–F) Illustration of the clinical set‐up of ultrasound, EMG and MMG to verify muscle responses when applying lumbar tSCS. (G) Full immersive environment by FES‐cycling on a road—seen in the background of the illustration. The participant sees his hands on the bicycle handlebar. EMG, electromyography; MMG, mechanomyography; tSCS, transcutaneous spinal cord stimulation. [Color figure can be viewed at wileyonlinelibrary.com]

In clinical practice, it is of the utmost importance to ascertain the efficacy of the treatment from the perspective of both the practitioner and the patient, and to ensure that it can be readily discerned and monitored. Hence, it is of great importance to monitor the efficacy of therapeutic interventions to ensure that the desired result is achieved without any evasive movements. This is because the spinal cord not only has a memory, but it is also capable of learning [31]. Clinical monitoring is indispensable, but insufficient for the purposes of planning the subsequent stage of treatment or checking the target site, whether in structural or functional terms. Technologies such as ultrasound imaging are useful for documenting structural features, including muscle thickness, pennation angle [32], the presence of fat and connective tissue, and contractility (see Figure 3A,B). Surface electromyography (EMG) is a valuable tool for examining muscle activation in a temporally accurate sequence. Furthermore, EMG can detect activity that is not directly related to the examined function and thus indicate unwanted muscle activity that is not part of the examined movement pattern. In addition to its diagnostic capabilities, EMG can also be employed as a feedback system in treatment, assisting the individual in learning the targeted muscle activity. In the future, additional diagnostic procedures may be employed, including mechanomyography (MMG) and near‐infrared spectroscopy (NIRS). MMG can be utilized, for instance, to discern muscle activity during stimulation, while functional NIRS can be deployed to visualize active regions on the cortex to document alterations after repeated utilization.

When utilizing tSCS, accurate electrode placement is essential for targeting specific muscle groups, such as the anterior/dorsal thigh muscles or the anterior/dorsal lower leg muscles. The combination of ultrasound, MMG, and EMG has been demonstrated to be an effective approach in this context (Figure 3C–F). It is, however, not imperative to utilize all of these methodologies. If one is accessible in a clinical setting, it confers an additional advantage to the treatment. Clinical observations by one of the authors (IB) have demonstrated that when tSCS is applied to the lumbar region, contractions in the lower limb musculature cannot always be palpated, but muscle activity could be detected in both the EMG and MMG signals as well as by the ultrasound imaging.

In addition to the various modalities of electrical stimulation, i.e., application sites, stimulation protocols, and paradigms, immersive learning strategies can be used in combination with virtual realities for treatment. The effect on motor learning using virtual environment scenarios is not inferior to that of motor learning in the real environment [33]. The spectrum and possibilities in clinics vary and can range from full immersion to the use of video games on a screen of varying sizes (Figure 3G).

The use of technology in therapy cannot and should not be based on the specific expertise of a single professional group. Frequently, the knowledge of therapists and clinicians, for instance, is insufficient to perform the necessary measurements and analyses. Conversely, engineers and scientists often develop technical methods that are not directly applicable in a clinical setting and do not address the specific clinical needs. The presence of a multidisciplinary team in the clinic is beneficial for the treatment and rehabilitation of individuals with neurological diseases. In conclusion, these technologies encompass diagnostic and therapeutic procedures that can be readily integrated with conventional methodologies. They enhance the precision of diagnostics, prognostics, and treatment by furnishing supplementary data on body structure and function.

## 4 BCI and FES

Since initial studies suggested that BCIs [34] may be used to induce beneficial neuroplasticity [35, 36], the number of clinical trials has increased dramatically (for recent reviews, see [37, 38, 39, 40]). BCIs enable the control of external devices through brain signals without relying on conventional muscle and nerve pathways [31], making them an ideal tool to integrate with other rehabilitation methods, such as FES.

In BCIs designed for inducing neuroplasticity, the user is asked to produce voluntary brain activity by attempting to perform or imagine performing a motor task. Once the user's intention is detected, an external device is activated to produce the intended movement, thereby closing the loop between brain activity and peripherally generated movement. These BCIs empower patients to take an active role in their recovery process and have led to significant and clinically relevant functional improvements in both upper and lower limb muscles (for reviews see [37, 38, 39, 40]). However, the BCIs vary in terms of the brain signals used for control, the specific devices (e.g., robotic actuators, electrical stimulators) employed, and the training paradigms applied (for recent review see [39]).

We recently developed and tested a BCI system designed to model PAS [41]. In our system, we detect the user's intent by extracting the movement‐related cortical potential (MRCP). The MRCP is a slow negative potential related to cue‐based or self‐paced motor task initiation [42, 43] that develops approximately −1630 ± 309 ms prior to attaining a peak negativity (PN) at which time the movement is executed [44]. At the time of PN, the motor cortex is active, sending the signals to the muscles to perform the planned action. In this sense, the MRCP PN may be viewed as a natural activation of the motor cortex compared to the TMS used during PAS, which activates the motor cortex in a very synchronous and artificial manner. Detection of the MRCP is used to trigger a stimulator to provide a single electrical stimulus to the target nerve at motor threshold intensity. This is timed in such a manner that the generated afferent volley arrives at the motor cortex during the PN of the MRCP. PAS and our associative BCI exhibit long‐term potentiation (LTP) and long‐term depression (LTD)‐like properties [41, 45, 46, 47]. LTP and LTD are mechanisms responsible for changes in synaptic strength that can persist for hours to days and are recognized as essential to learning [48, 49] and neuroplasticity [50, 51, 52], where the close timing of neuronal activations leads to strengthened synaptic connections. This synchronization promotes the likelihood of recurrent activations, which increases further with repetition.

In studies on lower limb function, associative BCI training has shown positive effects in chronic and subacute stroke patients with lower limb weakness [53, 54]. Peripheral stimulation of the deep branch of the common peroneal nerve was timed to coincide with the motor cortex's peak activity as patients attempted to perform dorsiflexion of the affected side. Results showed stronger corticospinal excitability, evidenced by significantly larger motor‐evoked potentials (MEPs) in the tibialis anterior muscle post‐intervention, translating into improved clinical function and walking distance, outcomes that were not observed in the control group.

In a series of studies in healthy participants, we have worked towards further refining our associative BCI and could show that both passively induced movements using a robotic actuator or FES‐generated muscle contractions as part of our associative BCI could induce similar neuroplastic changes when compared to the single ES [55]. These encouraging results now need to be tested on patient populations.

## 5 Sensorimotor Assessments

The study of pathological and healthy human motor control provided for decades important insights on how humans control their limbs. However, the clinical translation of these findings is still in its infancy, especially in the treatment of patients with limb loss. For example, goal‐directed movements such as reach‐to‐grasp tasks are well understood from the biomechanical, neuromuscular, and neurophysiological point of view [56]. Sensorimotor information is known to be integrated in a near‐optimal fashion following a Bayesian framework [57, 58]. The control of balance is a well‐studied neuro‐mechanical problem in healthy able‐bodied individuals [59]. Impairments in the control of our limbs such as those following central paralysis or limb amputation have also been vastly characterized in the literature. Gaps still exist; however, as in the case of neuromuscular adaptations following a lower limb amputation [60].

This accumulated knowledge is currently by far underused in the diagnostic and treatment protocols implemented in clinical settings. Remarkably, this knowledge has been poorly incorporated in the development and assessment of assistive devices such as bionic limbs [60]. The question is why this disconnection exists between basic knowledge of human motor control and the development of tools to assess and treat neurologically impaired individuals. The answer might be that the problem has two sides: the individuals generating/having this knowledge (scientists from physiology, movement sciences, motor control and engineering) and individuals in direct contact with the patients (physicians and physiotherapists) or directly designing assistive devices (prosthetists) do not work in mixed teams or do work in completely separate, non‐intersecting environments. The little investment from both sides toward finding a trade‐off between innovative implementation and realistic outcomes, namely patient progress and financial solvency, may halt optimal outcomes.

**FIGURE 4:**
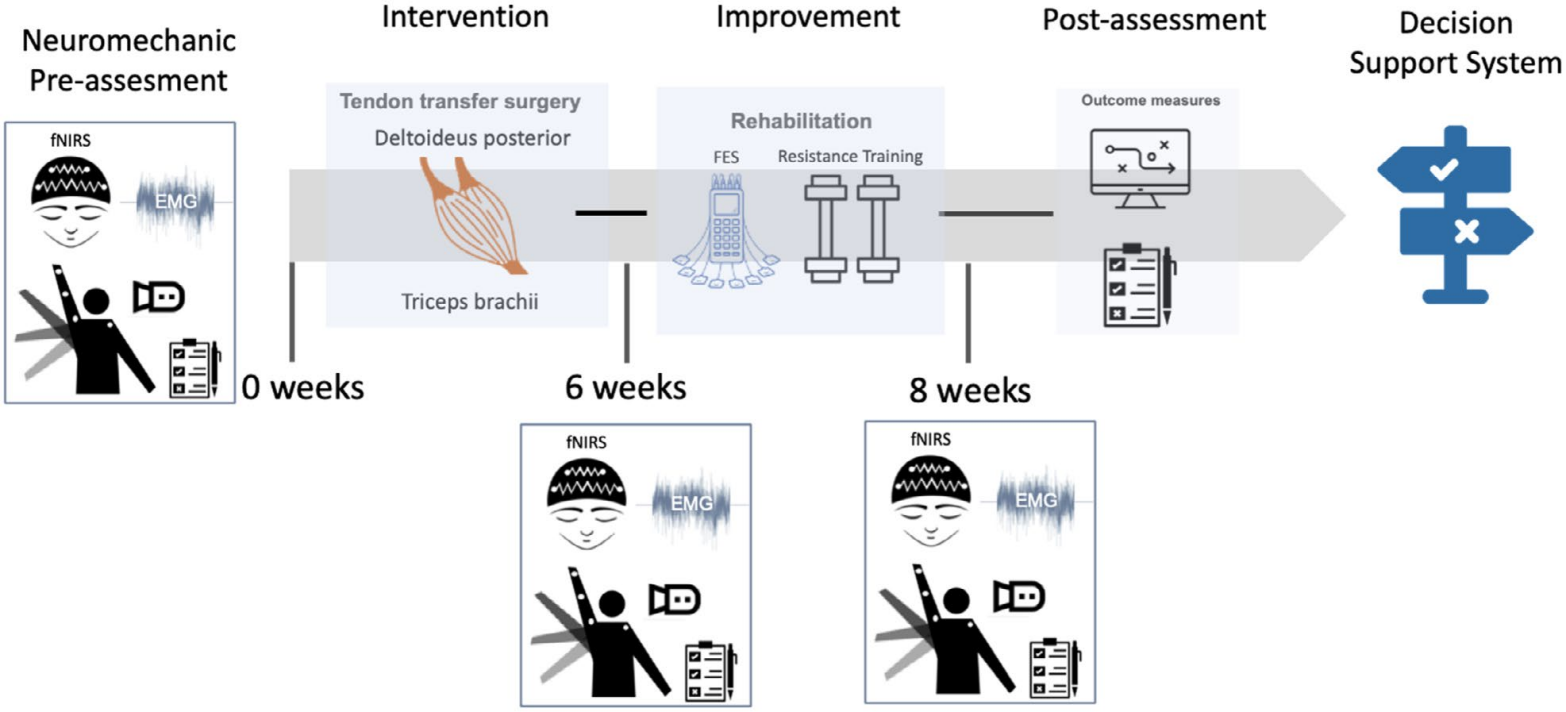
Possible workflow to assess motor control pre‐ and post‐intervention to facilitate candidate screening and outcome evaluation. [Color figure can be viewed at wileyonlinelibrary.com]

Possible direct translation includes the use of objective measures during goal‐directed movements such as tracking of kinematics variables (e.g., join angle variability, smoothness and velocity profiles) of impaired and intact limb segments, as well as intermuscular and corticomuscular coupling during the production of these movements. These relatively simple tasks facilitate the monitoring of the neuromuscular status of the patient, which may in turn facilitate the diagnosis, treatment, and objective evaluation of novel assistive devices such as limb prostheses (Figure 4). In the former case, it may help screen the patient's candidacy for surgical interventions. In the latter case, it may become essential to develop prosthetic devices that deliver sensory feedback via direct interfacing of the peripheral nervous system [60].

With the recent explosion in the use of inertial measurement units and other wearable technologies, the assessment of movement (independent of the neurological background) can be achieved at very low costs, limited only by the degree of education and specialization of the individuals in charge of the assessments. An open question remains on how to improve and bridge the gap between what we know about the neural basis of the human sensorimotor system and the direct treatment of patients with neurological disorders.

## 6 Open Topics and Future Directions

**FIGURE 5:**
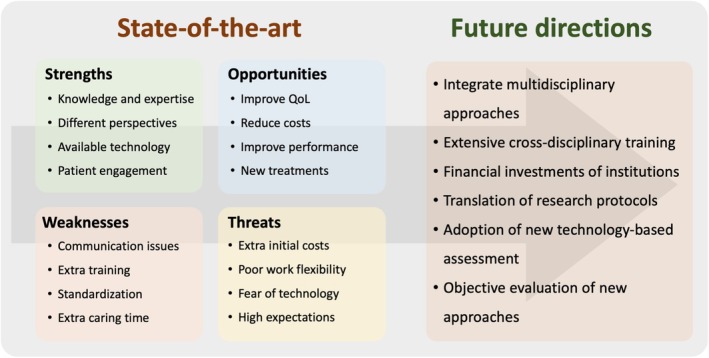
Analysis of current status and future directions of FES systems and BCI and motor assessment to close the gap in clinical establishment of comprehensive assessment. left: SWOT (strengths/weakness/opportunities/threats) analysis; right: Suggestions on the way forward. [Color figure can be viewed at wileyonlinelibrary.com]

We reviewed current non‐invasive technologies and methods that promise to enhance medical treatment and diagnosis using FES, BCI, and motor control assessments (Figure 5). These approaches have the potential to complement and enhance standardized clinical assessments with patient‐specific and personalized diagnostic and therapeutic interventions. A considerable raise in curiosity, awareness, and trust in these technologies is essential to achieve objective data beyond experience‐based subjective scales. Moreover, all medical practitioners involved in the diagnosis and treatment of patients with injuries of the nervous system must extend their scope of practice beyond the conventional boundaries of their roles as healthcare professionals. This requires assessment of movement beyond standardized clinical tests. Educational training and introduction to novel strategies and paradigms (function vs. device) is mandatory for achieving these goals. This would also include sales personnel and companies offering these technologies.

The authors experienced that clinical disciplines often operate in a highly specialized and isolated manner. Despite differences in application scenarios, similar devices are frequently employed across various fields. Electrodes, for instance, need to meet the same requirements for technical safety, independent of the purpose of stimulation. Key parameters such as material composition, electrode size, and stimulation protocols exhibit significant similarities across different and relatively independent use case scenarios and treatment approaches in paralysis, stroke, or following amputation. FES‐based paradigms on neural plasticity have been developed quite independently in the areas of paralysis after spinal cord injury, stroke, and after amputation. A comprehensive analysis and comparative evaluation of FES effects across these conditions may reveal underlying common mechanisms, facilitating the transfer of effective methodologies from one domain to another. For instance, BCI applications for synchronizing sensory feedback or monitoring the effects of specific stimulation protocols following amputation remain largely unexplored but could benefit from insights gained in stroke rehabilitation research.

There seems to be a distorted perspective between caregivers and individuals with technical backgrounds, with the first ones assuming a lack of practice in the clinical environment and an approach extremely “theoretical”, and with the second ones criticizing the lack of fundamental technical knowledge in “daily practice”. These two worlds may actually be closer to each other when improving communication and language to realize a true interdisciplinary teamwork. The end user requires technology that is straightforward and meaningful, which should provide them with enhanced functionality and/or increased independence.

It should be recognized and accepted that FES is quite often not a standalone therapy. Other treatments such as tDCS [61] and TMS [62] have short setup times to meet the window of opportunity in which success with respect to the outcome might be optimal. Time lines for non‐invasive BCI setup should be as short as for any other methodology. Setup of implantation of a iBCI or BMI (based on availability of such a system and availability of medical experts and surgical capacity) would probably exceed the timelines of the window of opportunity. Combination of therapeutic approaches should consider the cumulative rehabilitation impact to achieve best possible outcome. Opinion whether “more is better” from a theoretical point of science need to be very well balanced with “more practical” and comprehensive approaches in which medical care givers prefer to simplify the setup.

Regulation of medical devices is a concerning topic, especially in Europe. Another concern is the time required to perform novel diagnostics and treatments with technology that might require extra hours of data post‐processing before delivering reports for clinicians and physiotherapists. Clinical practice also requires pragmatic approaches to solve daily issues, as in the case of reusable electrodes. For example, a traffic‐light scheme would help to quickly visualize when an electrode should be disposed of. There also seems to be misperceptions about motor learning during FES application and a poor understanding of the effects of neuromuscular electrical stimulation (NMES), with the tradeoff of effect vs. affordability. Different wording of electrical stimulation (FES, NMES, TENS) raises quite often more confusion than benefits that detailed terminology would give. Funding seems further to be a limiting factor to implement technology in physiotherapy at the most optimal time for the patient.

## 7 Conclusions

We presented here a summary of the key points discussed at a scientific workshop at the 2024 Annual Conference of the International Functional Electrical Stimulation Society (IFESS) at the University of Bath (Bath, UK). The goal of this review was to expose the differences and commonalities in applying FES for the diagnosis and treatment of different neurological diseases. It is our intention that the aforementioned descriptions of the utilization of technology, technical expertise, and clinical knowledge to optimize patient care will provide motivation for the incorporation of novel assessment and treatment tools when employing FES in clinical settings. In a spirit of optimism and forward‐thinking, we encourage professionals from a range of research and clinical backgrounds to collaborate in a new era of objective measurement of patient improvement and clinical decision‐making.

## Author Contributions

All authors worked jointly on the concept and design of this article, which is an outcome of a workshop on Sept. 1st, 2024, at the IFESS conference in Bath (Bath, UK). All authors contributed to the introduction, open topics, and future directions, as well as the conclusions paragraph. T.S.: electrodes, I.B.: Motor learning, N.M.‐K.: BCI and FES, C.P.: Sensorimotor assessment.

## Conflicts of Interest

The authors declare no conflicts of interest.
